# Mapping the Geographical Distribution of Lymphatic Filariasis in Zambia

**DOI:** 10.1371/journal.pntd.0002714

**Published:** 2014-02-20

**Authors:** Enala T. Mwase, Anna-Sofie Stensgaard, Mutale Nsakashalo-Senkwe, Likezo Mubila, James Mwansa, Peter Songolo, Sheila T. Shawa, Paul E. Simonsen

**Affiliations:** 1 School of Veterinary Medicine, University of Zambia, Lusaka, Zambia; 2 Department of Veterinary Disease Biology, Faculty of Health and Medical Sciences, University of Copenhagen, Copenhagen, Denmark; 3 Center for Macroecology, Evolution and Climate, Natural History Museum of Denmark, University of Copenhagen, Copenhagen, Denmark; 4 Ministry of Health, Lusaka, Zambia; 5 World Health Organization, Regional Office for Africa, Harare, Zimbabwe; 6 University Teaching Hospital, University of Zambia, Lusaka, Zambia; 7 World Health Organization, Lusaka, Zambia; Centers for Disease Control and Prevention, United States of America

## Abstract

**Background:**

Past case reports have indicated that lymphatic filariasis (LF) occurs in Zambia, but knowledge about its geographical distribution and prevalence pattern, and the underlying potential environmental drivers, has been limited. As a background for planning and implementation of control, a country-wide mapping survey was undertaken between 2003 and 2011. Here the mapping activities are outlined, the findings across the numerous survey sites are presented, and the ecological requirements of the LF distribution are explored.

**Methodology/Principal findings:**

Approximately 10,000 adult volunteers from 108 geo-referenced survey sites across Zambia were examined for circulating filarial antigens (CFA) with rapid format ICT cards, and a map indicating the distribution of CFA prevalences in Zambia was prepared. 78% of survey sites had CFA positive cases, with prevalences ranging between 1% and 54%. Most positive survey sites had low prevalence, but six foci with more than 15% prevalence were identified. The observed geographical variation in prevalence pattern was examined in more detail using a species distribution modeling approach to explore environmental requirements for parasite presence, and to predict potential suitable habitats over unsurveyed areas. Of note, areas associated with human modification of the landscape appeared to play an important role for the general presence of LF, whereas temperature (measured as averaged seasonal land surface temperature) seemed to be an important determinant of medium-high prevalence levels.

**Conclusions/significance:**

LF was found to be surprisingly widespread in Zambia, although in most places with low prevalence. The produced maps and the identified environmental correlates of LF infection will provide useful guidance for planning and start-up of geographically targeted and cost-effective LF control in Zambia.

## Author Summary

Lymphatic filariasis (LF) is a debilitating mosquito borne parasitic infection which worldwide affects more than 120 million people. It is also widespread in Sub-Saharan Africa. A World Health Organization coordinated Global Programme to Eliminate LF has targeted LF for elimination as a public health problem by the year 2020, with annual mass drug administration (MDA) being the primary measure for this endeavor. An important first step before initiating MDA is the geographical mapping of infection in order to delimit the target areas. Past case reports have indicated that LF occurs in Zambia, but knowledge on its distribution and prevalence has been limited. Here we report on a country-wide survey carried out to map the geographical distribution and prevalence pattern across Zambia by screening adult volunteers for specific circulating filarial antigens (CFA). The CFA prevalences observed at the numerous survey sites are presented and mapped to give an indication of LF distribution in the country. The observed geographical variation is furthermore examined using a species distribution modeling approach to explore environmental requirements for LF presence, and to predict potential suitable habitats over unsurveyed areas. The findings provide a firm background for planning and start-up of LF control in Zambia.

## Introduction

Little has been reported about lymphatic filariasis (LF) in Zambia in the past. According to Buckley [1], local medical reports from the 1930's and 1940's mentioned the recovery of microfilariae (mf) of *Wuchereria bancrofti* from patients in Zambia, but the history and movements of the infected individuals did not rule out the possibility that infections had been acquired elsewhere. These reports also mentioned that the condition of elephantiasis was seen in Zambia and was commonly referred to as “Serenje leg” or “Feira leg” after its frequent occurrence in the districts of Serenje and Feira (now Luangwa). In 1946, Buckley identified a few cases of *W. bancrofti* microfilaraemia in hospital patients in Lusaka, Ndola and Kasama, but none of the infected individuals had been permanent residents in the country [1]. During a small night blood survey carried out in Luangwa valley, Barclay [2] failed to identify *W. bancrofti* mf. In contrast, both Buckley and Barclay reported high prevalences of infection with another human filaria, *Mansonella perstans*, from their surveys.

The first definite autochthonous case of LF due to *W. bancrofti* in Zambia was reported in 1975 by Hira [3], [4] from a 25-year old fisherman from Luangwa who presented with a tender swelling in the right inguinal fossa and swollen ankles. Hira [4], [5] afterwards observed more patients with *W. bancrofti* mf in Zambia, including cases acquired locally as well as cases that could have been acquired in neighboring countries. More recently, *W. bancrofti* mf were also reported from a 22-year old male from Southern Province [6] and from a 49-year old female from Northern Province who suffered from lower limb and vulval elephantiasis [7].

Although these observations suggested that LF was present and transmitted in Zambia, the geographical distribution, extent and prevalence pattern was largely unknown. In support of the World Health Assembly resolution of 1997 to eliminate LF globally as a public health problem, the government of Zambia therefore undertook a large-scale LF mapping survey from 2003 to 2011. Volunteers from all districts of the country were examined for circulating filarial antigen (a marker of *W. bancrofti* adult worm infection) according to guidelines from the World Health Organization [8]. A first objective of this paper is to outline the LF mapping survey activities and to empirically present the CFA prevalences as observed at the numerous survey sites across Zambia.

The presence of LF in an area is closely linked to the presence and abundance of the vector mosquitoes and to the physical requirements for parasite development within the vectors. Environmental conditions related to suitable mosquito habitats and to parasite growth and maturation in the vectors will often strongly influence the observed geographical prevalence patterns of LF [9], [10]. The environmental drivers of LF distribution can be explored through spatial modeling frameworks, and can in turn be used to predict parasite presence at unsurveyed locations to further guide control programmes. A second objective of this paper is to take advantage of the large dataset available from the mapping survey to identify the most important ecological correlates associated with LF infection and to use these to produce maps delineating the presence of LF at different prevalence levels in Zambia.

## Methods

### Ethical statement

The field surveys were carried out as a part of the Zambian Ministry of Health (MoH) Lymphatic Filariasis Control Programme (2003–2005) and Programme for Integrated Control of Neglected Tropical Diseases (2009–2011), and followed protocols approved by the MoH for these programmes. The selected survey populations were called for meetings during which they were given detailed information about LF and the background, purpose and implications of the survey. Individuals volunteering to be examined provided oral informed consent under observation of both project staff and village authorities (parents/guardians consented on behalf of children below 15 years). Oral consent is the traditional way for making agreements in the survey areas, where written consent is unfamiliar and would cause suspicion and refusal to participate.

### Selection of survey sites

All 72 districts of Zambia existing at the start of the activity in 2003 (some have later been split and/or reorganized) were targeted for LF mapping. Based on previous reports and hospital records indicating possible cases of LF, 14 districts located in eight provinces were first selected. These were Choma and Sinazongwe (Southern Province), Mpongwe (Copperbelt Province), Kalabo, Sesheke and Senanga (Western Province), Mbala and Chinsali (Northern Province), Chama and Lundazi (Eastern Province), Luangwa and Kafue (Lusaka Province), Serenje (Central Province) and Zambezi (North-Western Province). In each of these districts, three chiefdoms were selected to provide 100 volunteers each to be tested for circulating filarial antigen (CFA) during 2003–2005.

In the remaining 58 districts, which were considered less likely to have LF, one site was identified for the mapping exercise and 100 volunteers were targeted for CFA testing at each site during 2009–2011. Selection of the sites was facilitated by local health personnel who led the survey team to areas where the population of people was high enough to allow the required number of people to be tested.

### Field survey methodology

Members of the community were usually called to one central place for the CFA test. A clinic or health centre was found to be convenient for the purpose. Local health personnel were requested to assist in the exercise, and their presence brought confidence and trust, or less suspicion, from the community members. Geographical coordinates (longitude, latitude and elevation) were taken at the survey sites using a hand held GPS receiver (eTrex Summit, Garmin Corporation, Taiwan).

Following WHO guidelines [8], [11], about 100 volunteers above the age of 15 years were tested for CFA from each survey site. At few sites, however, volunteers down to the age of 12 years were allowed to participate due to low numbers coming forward for the test. From each individual, 100 µl finger-prick blood was collected using a heparinized capillary tube. The blood was applied to the specimen pad of a rapid immunochromatographic test card (ICT card, Binax Inc., USA). The result was read as positive or negative ten minutes after the card was closed and was recorded on a survey form together with the name, sex and age of the volunteer. The data were entered in Excel, and later transferred to SPSS for exploratory analysis.

### Environmental data

Proxy environmental variables that may potentially influence the distribution of the filarial parasite-host-mosquito system and hence LF transmission [9] were extracted from freely accessible Remote Sensing (RS) sources at spatial and temporal resolutions shown in Table 1. Daytime land surface temperature (LST day), night time land surface temperature (LST night) and the Normalized Difference Vegetation Index (NDVI) were averaged over the period 2001–2010 representing the climatic period of the LF survey, according to Zambia's three distinct climatic seasons: i) cold/dry season (May–August), ii) hot/dry season (September–November) and iii) hot/rainy season (December–April). Land cover data contained 23 different land cover classes for Zambia, which were re-classified into 7 categories; water bodies, wetlands, forests, urban areas, shrublands, grasslands and croplands and re-sampled to 1 km resolution. Rainfall estimates averaged over the climatic normal period 1950–2000 were obtained from the Worldclim project [12]. As a proxy for changes in the environment due to anthropometric activities, the human influence index (HII) [13] was used. A selection of maps of environmental predictors can be seen in Figure S1 in the supplementary material.

**Table 1:** Properties and sources of the remotely sensed and other environmental predictors used to model LF prevalence in Zambia.

Data type	Spatial resolution	Time period	Source
Day land surface temperature (LST day)	1×1 km	2001–2010	MODIS/Terra^1^
Night land surface temperature (LST night)	1×1 km	2001–2010	MODIS/Terra^1^
Normalized Difference vegetation Index (NDVI)	250×250 m	2001–2010	MODIS/Terra^1^
Land cover	1×1 km	2005	GLCN^2^
Water bodies (lakes and wetlands)	1×1 km	2005	GLCN^2^
Rainfall	1×1 km	1950–2000	WorldClim^3^
Altitude (DEM)	1×1 km	-	USGS^4^
Human Influence Index (HII)	1×1 km	-	SEDAC^5^

1 Moderate Resolution Imaging Spectroradiometer (MODIS); available at https://lpdaac.usgs.gov/ (accessed February 2012).

2 Global Land Cover Network (GLCN); available at http://www.glcn.org/databases/lc_gc-africa_en.jsp (accessed February 2012).

3 World Clim - Global Climate data, available at http://www.worldclim.org/ (accessed February 2012).

4 United States Geological Services (USGS) Digital Elevation Model (DEM) available at: http://eros.usgs.gov/ (accessed February 2012).

5 Socioeconomic Data and Applications Center, available at http://sedac.ciesin.columbia.edu/data/set/wildareas-v2-human-influence-index-geographic. (accessed February 2012).

The MODIS Reprojection Tool (USGS) was used to convert the RS data to geo-referenced maps. Further processing of the environmental data and distance calculation to the nearest water bodies was carried out in ArcMap v. 10.0 (ESRI). Additional data processing was performed in Revolution R Enterprise version 4.0 (Revolution Analytics; Palo Alto, USA) and Stata/SE 10 (StataCorp LP; College Station, USA). To elucidate potential co-linearity among the environmental variables, a correlation (Pearson's test) matrix was constructed based on 10,000 randomly extracted pixel values for each of the environmental predictors, with variables above a threshold of *r*>0.75 not allowed to enter the same model.

### Modeling approach

To explore the ecological niche of the LF parasite-vector-host biocoenose in Zambia, a species distribution modeling approach was deployed. Species distribution models, also referred to as ecological niche models [14], are commonly used to predict the geographic range of a species by extracting associations between point presence data and environmental data layers. The relationships are then used to characterize the environmental requirements of the species, and finally to predict suitable habitats across unsurveyed areas.

Here, species distribution modeling was implemented using the MaxEnt approach [15], commonly used to explore and predict environmental suitability for species, and has been shown to perform well compared to other predictive algorithms in comparative studies [16], [17]. A brief explanation of MaxEnt modeling is given in Methods S1 in the supplementary material. Recently, the MaxEnt approach has also been applied in mapping the Africa continent-wide current and potential future distribution of LF [18] and schistososmiasis [19]. Specifically, MaxEnt, which builds on the principles of maximum entropy, was chosen as it allows a flexible modelling of the often complex non-linear associations of infection presence with environmental variables [16], [18]. This flexibility can help facilitate an improved understanding of the ecological niche of a species, a prerequisite for a more reliable mapping of the potential distribution [20]–[22]. Furthermore, the MaxEnt method does not require absence data for the species being modeled; instead it takes advantage of the background environmental data for the entire study area through the background sampling procedure (see supporting information for more details). An advantage of this is dealing with the risk of including ‘false’ absence records in the model that can arise from limitation of parasite detectability [23] and hence falsely indicate non-suitability of a location. Finally, MaxEnt copes relatively well with correlated variables (which environmental variables often are) through the inbuilt method for regularization (L1-regularization) known to be well-performing [24], making it possible to explore a wider breath of potential environmental dependencies.

Two separate models were explored, based on different prevalence value cut-offs: Model 1 was based on survey sites that had at least 5% prevalence, and model 2 used survey sites with at least 15% CFA prevalence as MaxEnt model input data. This was done to get an indication of the drivers of both the general distribution of endemic LF in Zambia (represented by the distribution of at least 5% CFA prevalence), as well as the distribution of medium to high levels of infection prevalence (at least15% prevalence).

The spatial output of the MaxEnt model consists of a continuous range of relative probabilities indicating, in the case of this study, presence of the host–parasite system at the given prevalence threshold. The default logistic model that gives predicted estimates between 0 and 1 of the probability of infection presence for each pixel in the map was used. It was chosen to fit only linear, quadratic and product relationships, since more complex models can be difficult to specify a priori based on ecological theory [25]. Other parameterizations (maximum number of iterations and convergence threshold) followed recommendations by the model developers [15], [26].

The importance of the environmental variables was evaluated by comparing estimates of the relative contribution of environmental factors to overall model training gain. The gain is a measure closely related to deviance, the goodness of fit measure used in generalized additive and generalized linear models [15]. Furthermore, the explanatory information in each variable when used in isolation and the information lost when omitted from the model was quantified using a jackknife cross-evaluation procedure.

The continuous probability maps were furthermore converted into binary presence/absence maps of the LF host–parasite system, using the threshold indicating maximum training sensitivity plus specificity (i.e., that threshold which maximizes the sum of sensitivity and specificity for the training data). This is one of 11 thresholds calculated by MaxEnt, and is in considered one of the more robust of several standard thresholds for converting continuous probability surface to presence/absence surface [27], [28]. This distribution was then used to define the spatial limits for each of the two categories (≥5% and ≥15%) of infection prevalence.

### Model evaluation

A validation procedure was implemented by randomly dividing the occurrence data in training and test data sets (based on a 80–20% splitting of the data set). The evaluation focused on predictive performance at sites. Three statistics were applied; 1) the Area under the receiver operating characteristic Curve (AUC), 2) correlation (COR) and 3) sensitivity and specificity, to assess the agreement between the prevalence recorded at sites and the predictions.

AUC ranges from 0 to 1, where an AUC≤0.5 indicates that model performance is equal to or worse than that of a random prediction while an AUC above 0.75 is normally considered useful [17]. COR was calculated as the Pearson correlation coefficient between the full range of prevalence values in the test dataset (including negative sites) and the model logistic prediction [16], [26]. Sensitivity was calculated as the proportion of true positives/negatives (‘presence/absence’ points) falling within the predicted presence/absence area, and specificity as the proportion of true negatives falling within the predicted absence area.

## Results

### Study sites, study population and CFA prevalences

A total of 10193 volunteers from 108 survey sites located in all 72 districts and 9 provinces of Zambia were surveyed for CFA. Among these, 9964 (97.8%) had a valid test card result and comprise the study population of examined individuals analyzed in this study. An overview of the survey sites, and the number, positivity for CFA, age and sex of the study population, is presented in Table 2. A list of geographical coordinates for the study sites is given in Table S1 in the supplementary material.

**Table 2:** Overview of study sites, and the numbers, positivity for circulating filarial antigens (CFA), ages and gender ratios of examined volunteers.

Site no.	Province	District	Village/Chiefdom/Site	Altitude in m	Volunteers examined for CFA			
					No. Examined	No. positive (%)	Mean age (range) in years	Female∶male ratio
1	Central	Mkushi	Masansa	1267	102	3 (2.9)	32.1 (15–71)	1.37
2		Kapiri Mposhi	Tazara	1228	101	6 (5.9)	36.6 (16–65)	2.74
3		Chibombo	Chibombo	1068	100	3 (3.0)	42.2 (15–77)	0.96
4		Kabwe	Kasanda	1086	101	9 (8.9)	30.9 (16–60)	2.26
5		Mumbwa	Keezwa	980	102	8 (7.8)	26.1 (15–95)	1.00
6		Serenje	Mulilima	1464	95	0 (0.0)	26.0 (15–60)	8.50
7		Serenje	Muchinka	1430	100	16 (16.0)	37.4 (15–86)	0.85
8		Serenje	Mapepala	1160	101	20 (19.8)	29.9 (15–67)	1.97
9	Copperbelt	Mpongwe	Mwanankonesha/Lesa	1250	98	0 (0.0)	35.2 (15–83)	1.39
10		Mpongwe	Machiya	1149	102	0 (0.0)	30.8 (15–68)	1.04
11		Mpongwe	Mwinuna	1160	101	0 (0.0)	33.5 (15–70)	2.48
12		Masaiti	Fiwale Mission	1275	103	6 (5.8)	40.1 (15–86)	1.24
13		Ndola	Chipulukusu	1242	102	3 (2.9)	34.1 (15–70)	5.00
14		Luanshya	Mpatamatwe	1255	100	8 (8.0)	28.3 (15–68)	1.70
15		Kitwe	Buchi	1218	101	2 (2.0)	31.1 (16–73)	4.32
16		Chililabombwe	Kawama	1323	100	1 (1.0)	27.7 (15–62)	6.69
17		Lufwanyama	St. Joseph Mission	1220	100	10 (10.0)	30.9 (17–79)	1.22
18		Kalulushi	Chibuluma	1284	100	5 (5.0)	38.9 (15–88)	1.38
19		Mufulira	Lwansobe	1287	102	4 (3.9)	43.9 (15–85)	2.92
20		Chingoloa	Chawama	1362	102	2 (2.0)	34.9 (15–83)	2.13
21	Eastern	Chadiza	Nsadzu	296	101	0 (0.0)	23.7 (14–70)	1.15
22		Chipata	Madzimoyo	921	99	1 (1.0)	25.2 (15–75)	2.54
23		Mambwe	Masumba	557	101	2 (2.0)	33.3 (15–95)	2.26
24		Katete	Katete Urban	1025	101	1 (1.0)	33.0 (15–78)	1.02
25		Nyimba	Chipembe	857	105	0 (0.0)	33.3 (15–76)	2.62
26		Petauke	Mumba	989	102	1 (1.0)	28.5 (15–66)	6.85
27		Lundazi	Zumwanda	1133	103	7 (6.8)	34.0 (15–85)	1.34
28		Lundazi	Nkhanga	1092	102	11 (10.8)	37.0 (15–85)	1.17
29		Lundazi	Mwase-Lundazi	1215	106	17 (16.0)	39.0 (18–82)	1.26
30		Chama	Chipundu-Kambombo	733	81	0 (0.0)	32.5 (16–61)	1.89
31		Chama	Mbubeni-Tembwe	676	80	0 (0.0)	32.4 (18–74)	1.35
32		Chama	Chitunda-Chikwa	685	76	0 (0.0)	31.1 (19–73)	4.07
33	Luapula	Chiengi	Puta	970	38	0 (0.0)	32.8 (18–73)	1.92
34		Nchelenge	Nchelenge	924	99	0 (0.0)	29.9 (15–76)	4.50
35		Kawambwa	Mukamba	1201	45	1 (2.2)	30.6 (15–65)	1.65
36		Mwense	Lubunda	928	50	1 (2.0)	44.2 (18–75)	1.50
37		Mwense	Musangu	963	33	0 (0.0)	38.1 (17–68)	3.71
38		Mwense	Lukwesa	954	18	0 (0.0)	45.8 (24–79)	2.00
39		Mansa	Mabumba	1244	54	0 (0.0)	44.6 (16–82)	1.70
40		Samfya	Mandubi	1148	60	0 (0.0)	38.7 (20–71)	3.00
41		Milenge	Milenge East 7*	1196	106	22 (20.8)	41.2 (15–70)	1.36
42	Lusaka	Lusaka	Chipata	1249	103	0 (0.0)	30.9 (14–68)	5.87
43		Chongwe	Rufunsa	910	102	4 (3.9)	27.3 (15–78)	2.19
44		Kafue	Chanyanya Harbour	977	100	30 (30.0)	36.4 (15–91)	1.08
45		Kafue	Kanjawa	1211	100	14 (14.0)	36.0 (15–96)	1.70
46		Kafue	Tukunta	1153	100	12 (12.0)	31.1 (16–84)	6.14
47		Luangwa	Kavalamanja-Mphuka	377	91	33 (36.3)	29.9 (15–60)	1.39
48		Luangwa	Janeiro-Mphuka	349	100	33 (33.0)	27.4 (15–70)	1.44
49		Luangwa	Chitope-Mburuma	371	76	19 (25.0)	34.4 (16–69)	1.92
50	Northern	Luwingu	Nsombo	1175	100	11 (11.0)	34.3 (15–78)	1.70
51		Chilubi	Chaba	1189	100	11 (11.0)	36.8 (15–89)	1.13
52		Kaputa	Kalaba	944	104	6 (5.8)	26.2 (15–72)	0.79
53		Mporokoso	Chishamwanba	1424	100	5 (5.0)	28.6 (15–75)	1.22
54		Mpulungu	Mpulungu	778	102	10 (9.8)	30.7 (14–96)	3.25
55		Isoka	Kampumbu	770	101	8 (7.9)	36.9 (15–77)	0.98
56		Nakonde	Shemu	1341	98	7 (7.1)	34.4 (17–82)	0.56
57		Mungwi	Mumba	1212	101	6 (5.9)	33.7 (15–70)	1.59
58		Kasama	Munkonge	1255	99	6 (6.1)	32.2 (15–70)	1.15
59		Mpika	Nabwalya	549	100	3 (3.0)	22.0 (15–70)	0.75
60		Mpika	Mpepo	1257	92	3 (3.3)	23.6 (41–68)	0.96
61		Mbala	Chilundumusi	1383	101	0 (0.0)	29.9 (15–82)	1.30
62		Mbala	Mwamba	1567	99	0 (0.0)	27.6 (15–77)	0.98
63		Mbala	Chiungu-Zombe	1257	94	1 (1.1)	36.5 (15–87)	1.85
64		Chinsali	Ilondola-Nkula	1342	93	0 (0.0)	41.9 (13–85)	0.94
65		Chinsali	Nkweto	1292	89	0 (0.0)	26.8 (14–68)	1.78
66		Chinsali	Mulanga**	1268	73	0 (0.0)	21.2 (14–76)	0.74
67	North-Western	Mwinilunga	Kalene Mission	1195	100	1 (1.0)	39.0 (15–82)	1.27
68		Solwezi	Solwezi Urban	1336	100	2 (2.0)	30.7 (15–67)	1.86
69		Solwezi	Lumwana East	1273	106	3 (2.8)	33.5 (15–80)	2.53
70		Kasempa	Kasempa Urban	1220	101	5 (5.0)	32.8 (12–80)	1.89
71		Mufumbwe	Boma	1159	106	5 (4.7)	30.2 (15–72)	1.47
72		Kabompo	Kapompo	1127	102	2 (2.0)	46.0 (17–89)	1.00
73		Chavuma	Chiyeke	1075	103	5 (4.9)	36.8 (15–89)	1.15
74		Zambezi	Kucheka	1058	59	0 (0.0)	41.2 (15–95)	0.90
75		Zambezi	Mukandankunda***	1080	148	1 (0.7)	37.5 (15–88)	1.48
76		Zambezi	Chinyingi-Ndungu	1050	67	1 (1.5)	36.9 (15–75)	2.19
77	Southern	Livingstone	Lubuyu	864	100	2 (2.0)	33.5 (15–64)	4.26
78		Kazungula	Makunka	1036	99	6 (6.1)	31.6 (15–68)	1.68
79		Kalomo	Namiyanga	1252	100	4 (4.0)	32.5 (21–80)	2.57
80		Monze	Njola Mwanza	1026	99	6 (6.1)	32 9 (15–68)	11.4
81		Itezhitezhi	Itezhitezhi Urban	942	98	14 (14.3)	30.7 (15–61)	7.91
82		Gweembe	Munyumbwe	618	105	9 (8.6)	27.6 (14–60)	2.28
83		Siavonga	Siavonga District	510	101	3 (3.0)	31.3 (15–63)	1.59
84		Namwala	Muchila	1071	100	5 (5.0)	37.9 (15–71)	3.76
85		Namwala	Chitongo	309	64	9 (14.1)	29.6 (15–60)	1.29
86		Mazabuka	Cheeba	301	102	1 (1.0)	36.9 (15–87)	1.76
87		Choma	Simachenga-Singani	1289	99	1 (1.0)	32.5 (15–75)	2.96
88		Choma	Macha	1155	101	0 (0.0)	36.2 (15–73)	1.35
89		Choma	Moyo	1002	126	0 (0.0)	42.2 (16–83)	1.42
90		Sinazongwe	Sinazeze	625	85	5 (5.9)	39.4 (16–77)	1.43
91		Sinazongwe	Sinazongwe	492	98	5 (5.1)	40.2 (18–83)	2.27
92		Sinazongwe	Mwemba	497	93	0 (0.0)	36.2 (17–70)	2.32
93	Western	Kaoma	Mangango Mission	1127	39	1 (2.6)	37.2 (15–70)	2.55
94		Kaoma	Mayukwayukwa 1	1068	64	9 (14.1)	34.5 (15–79)	1.86
95		Lukulu	Silembe****	1058	98	2 (2.0)	41.8 (15–89)	1.23
96		Mongu	Nalikwanda*****	1049	51	1 (2.0)	42.9 (17–77)	0.82
97		Shangombo	Nangweshi	1022	83	8 (9.6)	33.8 (15–75)	1.44
98		Mongu	Sefula–Namutwe	1034	49	3 (6.1)	35.5 (17–60)	1.88
99		Kalabo	Maunyambo	1020	85	6 (7.1)	43.9 (13–81)	1.30
100		Sesheke	Mulundamo	952	100	6 (6.0)	41.5 (16–85)	2.45
101		Sesheke	Malabwe	929	99	1 (1.0)	39.7 (16–77)	4.67
102		Sesheke	Sazibilo	947	99	7 (7.1)	34.3 (16–86)	1.30
103		Senanga	Itufa-Lityamba	1024	94	28 (29.8)	34.7 (15–80)	2.24
104		Senanga/Shangombo	Kanja/Nangweshi	995	100	24 (24.0)	40.8 (15–78)	3.17
105		Senanga	Kaunga Lueti	1013	102	23 (22.5)	34.6 (16–78)	1.76
106		Kalabo	Nalubutu Sishekanu	1041	76	41 (53.9)	34.9 (15–79)	4.43
107		Kalabo	Kaonga Sikongo	1014	81	41 (50.6)	38.7 (15–80)	2.38
108		Kalabo	Lwandamo Lutwi	1046	91	48 (52.7)	40.0 (16–85)	2.64
All	-	-	-	-	9964	736 (7.4)	34.0 (12–96)	1.78

Only volunteers with a valid CFA test result are included (tests of 229 volunteers produced invalid results).

* Milenge East 7 & Changwe Lungo.

** Mulanga-Chibesakunda.

*** Mukandankunda-Ishindi.

**** Silembe Kalambwe-Imenda.

***** Nalikwanda–Singonda.

Most survey sites (83 or 76.9%) had more than 90 examined individuals, whereas 14 sites (13.0%) had less than 70. The highest mean number of examined individuals per site (100.9) was in Copperbelt Province, whereas the lowest (55.9) was in Luapula Province. The age of examined individuals ranged from 12 to 96 years. The mean age for the survey sites ranged from 21.2 to 46.0 years, and the overall mean age was 34.0 years. Many more females than males were examined (6376 vs. 3585), and the great majority of sites had more examined females than males (94 or 87.0%).

CFA positive cases were identified at 84 (77.8%) of the survey sites, where the prevalence ranged from 1.0 to 53.9%. The prevalence was ≥5% at 49 sites and ≥15% at 14 sites. The highest mean CFA prevalences were seen in Western (19.0%) and Lusaka (18.8%) provinces, whereas the lowest were in Copperbelt (3.4%) and North-Western (2.5%) provinces. The overall mean CFA prevalence for all examined sites was 7.4%.

**Figure 1:**
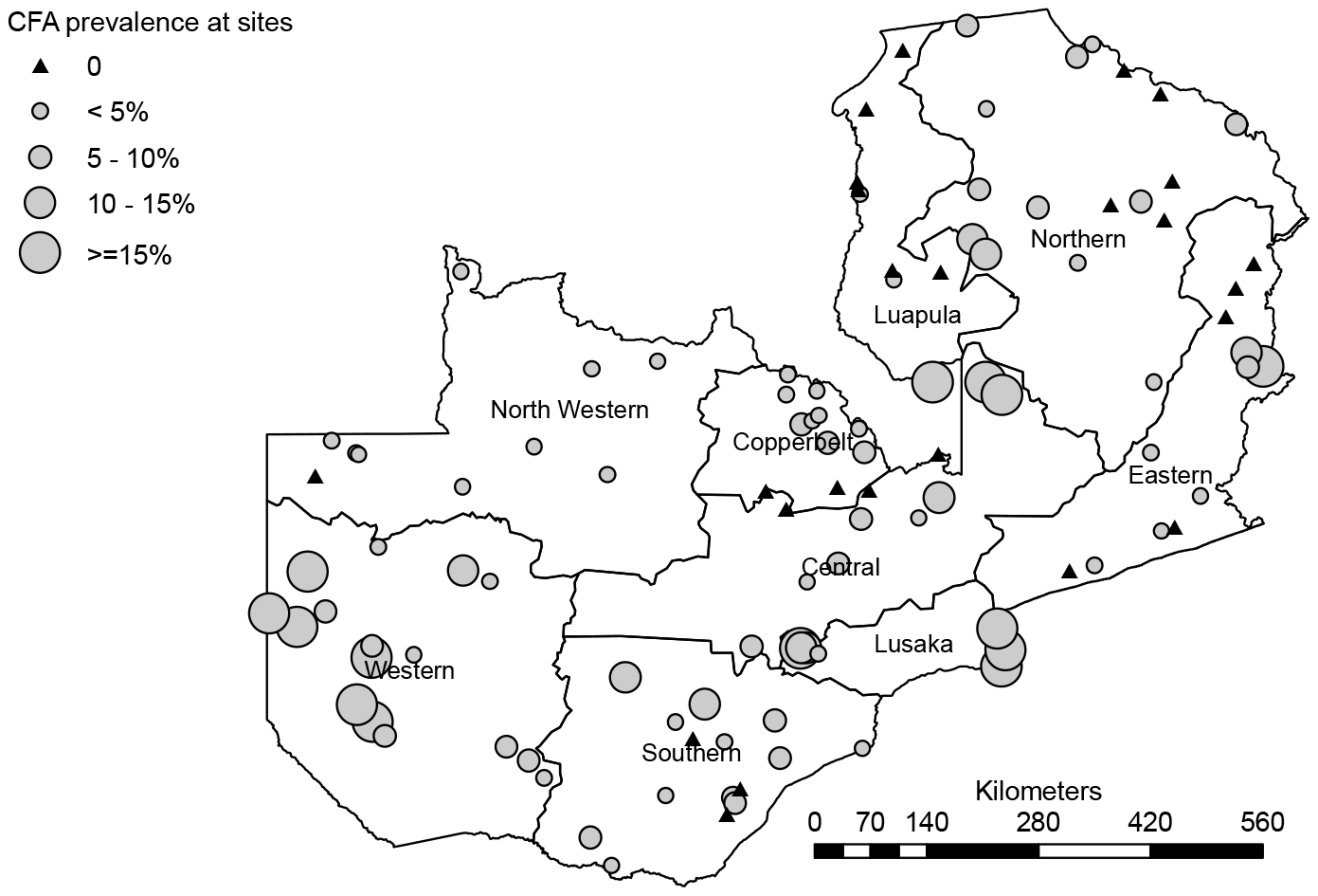
Map of Zambia showing survey sites and prevalences of CFA positivity.

A graphical presentation of the measured CFA prevalence at the different survey sites is shown in Figure 1, which thus gives an overview of the distribution pattern of LF in Zambia. All provinces had sites with a low CFA prevalence below 15%. However, six foci with CFA prevalences above 15% are clearly identified from the figure. Named by the district of location, these are the Kalabo and Senanga focus (both in Western Province), the Luangwa and Kafue focus (both in Lusaka Province), the Serenje focus (Central Province) and the Lundazi focus (Eastern Province). The first four of these foci had sites with particularly high CFA prevalences of >25%, and among these the Kalabo focus had sites with >50% CFA prevalence.

### Model outputs

The importance of the environmental determinants of LF distribution in Zambia, as measured by their contribution to overall model training gain, varied substantially between the model based on ≥5% and the model based on ≥15% CFA prevalence data. The relative contribution of the 7 most important (of a total of 16) of the environmental predictor variables is given in Table 3. Between them, these 7 predictors were ranked in the top three of at least one of the two models. Overall the most important predictor was land cover, which in particular for model 1 (CFA≥5%) contributed to a significant part of model gain. In particular croplands and grasslands were associated with high probabilities of presence of infection, whereas forested areas were predicted as the least suitable of the land cover classes. The second most important predictor variable was day land surface temperature (hot/dry season). It was especially important in the CFA≥15% model, where it contributed 22.4% of total training gain, which is in accordance with the jackknife procedure that indicated that it was also the variable with the highest model gain when used in isolation. The human influence index, HII, was also an important predictor in model 1 where it contributed 20.9% of total training gain, whereas it did not play a significant role in model 2, contributing only 1.5% of total training gain.

**Table 3:** Summary statistics of jackknife test of environmental variable importance, evaluation measures, and maximum training sensitivity plus specificity threshold results for MaxEnt model 1 (sites with CFA≥5%) and model 2 (sites with CFA prevalence ≥15%).

	Model 1 (CFA≥5%)	Model 2 (CFA≥15%)
**Variable contribution to model training gain (%)**		
Land cover	**34.3**	**23.8**
Human Influence Index (HII)	**20.9**	1.5
LSTday* (hot-dry season)	**19.6**	**22.4**
Distance to water bodies	6.1	11.7
NDVI** (hot-dry season)	5.4	1.0
LSTday (rainy season)	2.4	**13.7**
Altitude (DEM)	0.2	9.1
**Model evaluation measures**		
AUC (SD)***	0.866 (0.045)	0.892 (0.074)
CORprev**** (*p*-value)	0.117 (0.234)	0.355 (<0.001)
Threshold dependent sensitivity	68.8%	76.9%
Threshold dependent specificity	46.6%	64.5%
Threshold cut-off probability value	0.412	0.465

Only the 7 predictors that were ranked in the top three of at least one of the two models are included. The top three predictors for each model are highlighted in bold.

*LST; Land Surface Temperature.

**NDVI; Normalized Difference vegetation Index.

***AUC; the area under the Receiver Operating Characteristic curve (and standard deviation).

**** CORprev is the Pearsons product moment correlation between model logistic probability and the measured CFA prevalence at survey sites.

The least important environmental factors for both models, as judged from the total gain, were rainfall and night time LST. The environmental variable that decreased the gain most when omitted was the distance to surface water bodies, which therefore appeared to have the most information not present in the other variables.

**Figure 2:**
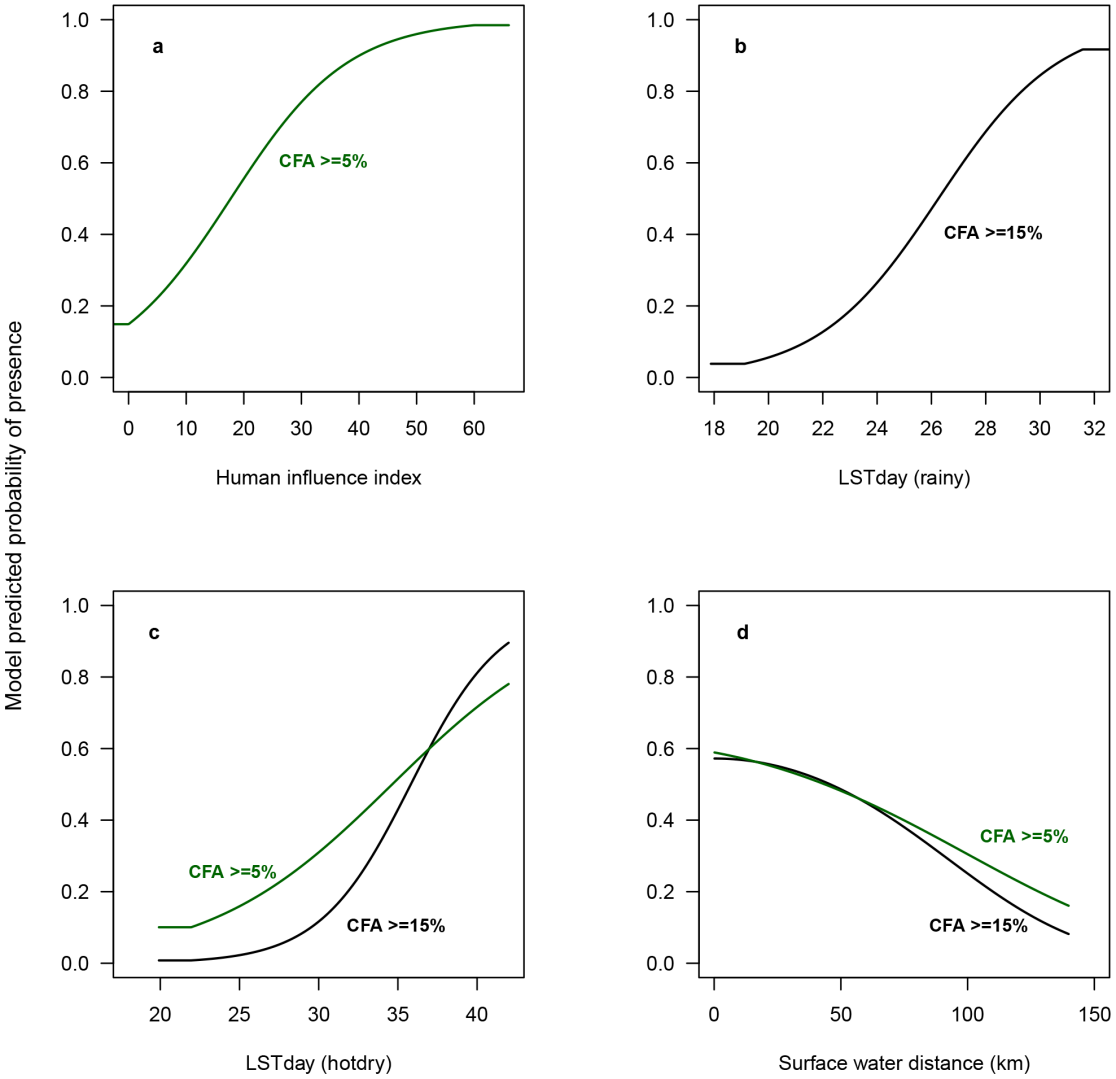
Response curves illustrating the relationship of MaxEnt predicted probability of occurrence to environmental variables. The values shown on the y-axis is the predicted probability of suitable conditions, as given by the logistic output format, with only the particular predictor variable used to develop the MaxEnt model. (a) The figure shows the relationship between the Human Influence Index and the predicted probability of occurrence of CFA≥5% (model 1), (b) depicts the relationship between day-time land surface temperature in the rainy season (LSTday (rainy)) and the probability of LF as modeled by model 2 (CFA≥15%), (c) shows the relationships between day-time land surface temperature in the hot-dry season (LSTnight (hot-dry) and the probability of LF occurrence as modeled by model 1 and 2, respectively, and (d) shows the relationship between the distance to nearest surface water bodies and the probability of occurrence of LF as modeled by model 1 and model 2, respectively.

The functional relationship between the most important continuous predictor variables and the predicted probability of presence of either ≥5% or ≥15% CFA is depicted in the response curves in Figure 2. Each curve is made by generating a MaxEnt model using only the corresponding predictor variable, disregarding all other variables.

**Figure 3:**
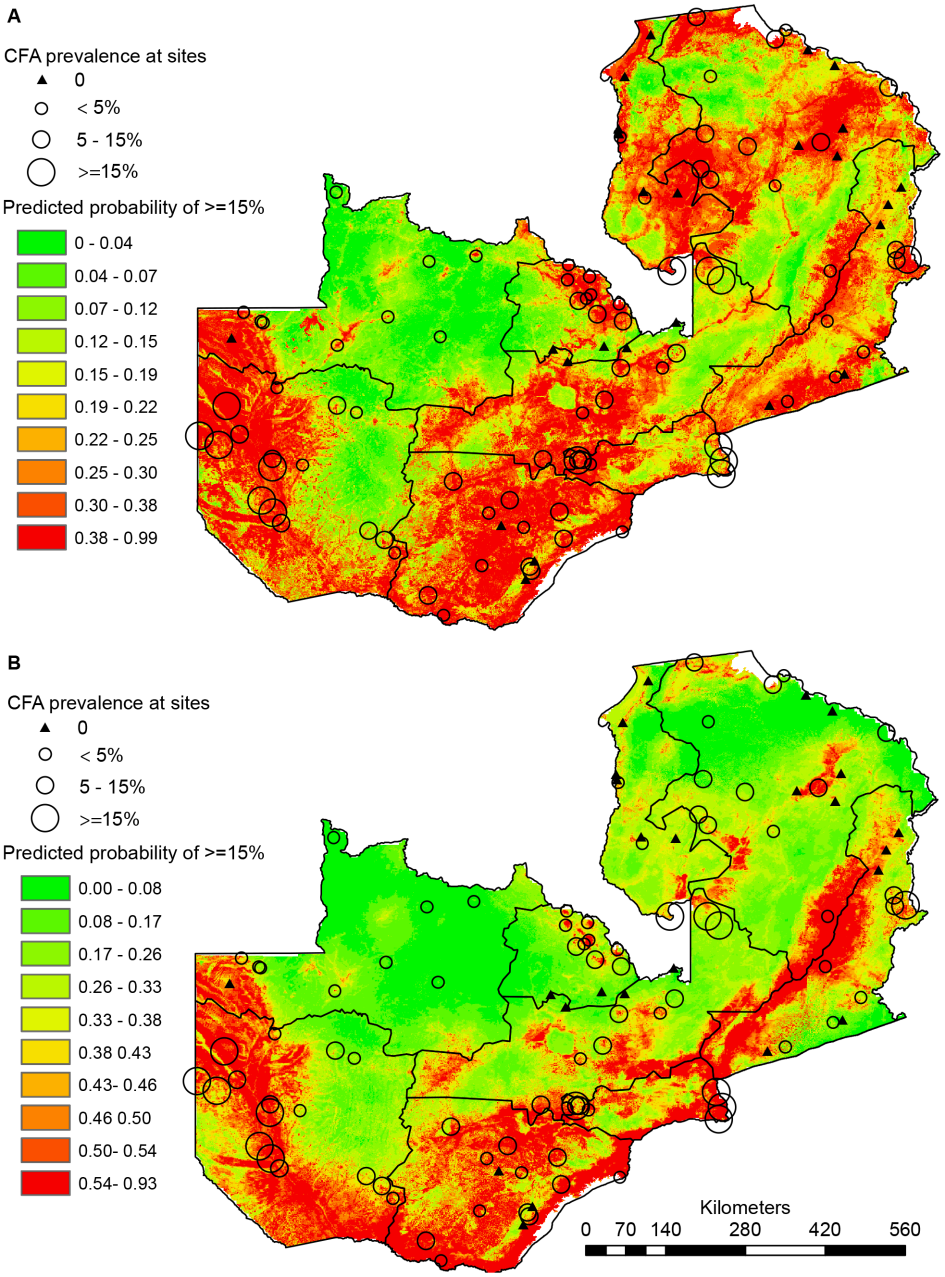
Maps of the MaxEnt predicted distributions of CFA prevalence categories. (A) The heatmap values represent the relative probabilities of presence of LF with at least 5%, CFA prevalence (model 1). (B) The heatmap represent the predicted relative probability of presence of LF with at least 15% CFA prevalence (model 2).

Maps of the MaxEnt predicted distributions of low (≥5% CFA) and medium-high LF infection prevalence (≥15% CFA) categories are presented in Figure 3a and 3b respectively. The heat map values represent the probabilities of ‘presence’ of each prevalence category, with relative probability values ranging from 0 (green colors) to 1 (red colors). The scale is defined for each map so that red areas correspond to ‘presence areas’ as defined by the threshold indicating maximum training sensitivity plus specificity.

Both maps indicate that LF infection potentially is present across Zambia with a somewhat patchy distribution, but with particularly high probability of presence in the floodplains of Western Province, the western part of North-western Province, the flood plain areas surrounding Zambezi River and its tributaries, the areas along Lake Kariba, the Kafue plains and the low plateau and river floodplains of Luangwa River. The most notable difference between the two maps is the much more confined presence areas predicted for the ≥15% prevalence category in the Northern and Luapula Provinces as compared to the relatively large areas predicted as potential ≥5% prevalence presence in these provinces.

**Figure 4:**
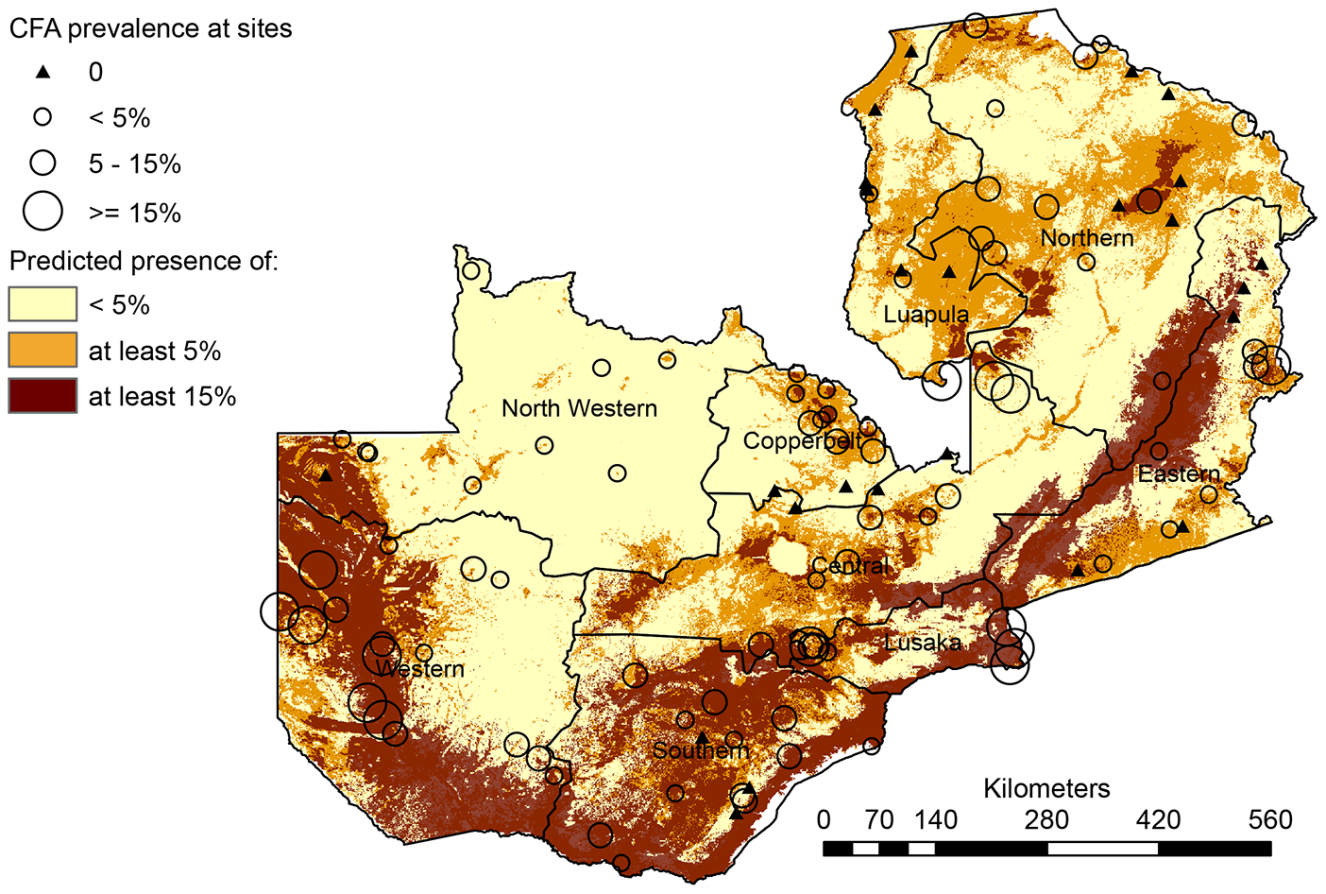
Map resulting from the overlay of the thresholded versions of the maps in Figure 4. The map depicts areas of predicted presence of ≥15% CFA prevalence (brown), ≥5% CFA prevalence (orange+brown) and areas where no or <5% CFA is predicted to be present (light yellow).

Superimposing the binary presence/absence maps to produce one risk map (Figure 4) furthermore highlighted differences and similarities between the two model predictions. The orange color in this figure represents areas where only model 1 (CFA≥5%) predicts presence and the dark-brown color shows where model 2 (CFA≥15%) predicts presence (nested within model 1 predicted presence areas). Finally, the light yellow color in the map delineates areas where none of the models predict presence, i.e. areas expected to have no or less than 5% infection prevalence.

### Model performance

Measures of model accuracy are presented in Table 3. AUC values ranged from 0.866 to 0.892, indicating that the ‘suitability’ for LF infection was correctly ranked for 87–89% of the evaluated map pixels.

The correlation (COR) between the MaxEnt model predicted suitability and the observed full range of CFA prevalences at all 108 localities ranged from 0.117–0.355, and increased with CFA prevalence cut-off level (Table 3). This indicates that MaxEnt modeled the ‘true’ prevalence pattern of LF infection in Zambia better when using medium to high prevalence localities only (model 2), rather than the more general presence of infection (≥5%) which showed a non-significant correlation to the observed CFA prevalences at survey sites. Based on the presence/absence map, model 2 also had the best predictive positive and negative performance as evaluated by its sensitivity (76.9%) and specificity (64.5%) meaning that 76.9% of the ≥15% prevalence data points were correctly identified within the predicted ‘≥15% prevalence zone’, and that 64.5% of the true negatives were correctly identified within the ‘<15% prevalence zone’.

## Discussion

The field survey reported in this paper was the first country-wide screening for LF in Zambia. More than 10,000 people from 108 sites located in all 72 districts and 9 provinces were examined for CFA during an 8-year period from 2003 to 2011. The survey surprisingly indicated that LF is widely distributed in the country, with 78% of sites having CFA positive cases. In many of the sites prevalences were rather low, but a few identified foci had prevalences above 25%. The highest prevalences (above 50%) were recorded from Kalabo District in Western Province. The results from the survey, in particular the identification of the high endemicity foci, provide an important background for planning and initial implementation of LF control measures in Zambia.

Females were much more eager to participate in the CFA screening than males. Overall, 64% of those examined were females, and at most survey sites (87%) more female than male volunteers were examined. It is well known that the LF prevalence in most endemic areas is higher in adult males than adult females [28]–[30]. The recorded prevalences from the Zambian survey may therefore be an underestimation of the true values, especially at sites where the female to male ratio was high. Similarly the potential sampling biases introduced by involving local health personnel in the selection of study sites (oversampling of suspected endemic areas) and by examination of volunteers (non-random sampling of study individuals) should be kept in mind when interpreting findings. These are, however, practical arrangements that are often difficult to avoid during large-scale mapping surveys, and which are also recognized in the WHO guidelines for mapping surveys [8], [11].

Some of the identified high prevalence foci were located near national borders, and it is possible these may be attached to foci in neighboring countries. Thus, the river Zambezi separates the Luangwa focus from areas of Zimbabwe where cases of LF have previously been documented [31], [32], and LF moreover appears to be common in the nearby Tete Province of Mozambique [33]. The Lundazi focus is close to Malawi, which also has widespread occurrence of LF although the prevalence in the western part of the country tends to be low [34]. Whether the Kalabo and Senanga foci extend into nearby Angola, or the Serenje focus extends into nearby Democratic Republic of Congo, is unclear as current information about the geographical distribution of LF in these neighboring countries is limited [33], [35]. Infections with another species of filarial parasite, *M. perstans*, have also been reported from humans in Zambia [1], [2], but these do not seem to cross react in the CFA tests for *W. bancrofti* [36].

Knowledge about the vectors of LF in Zambia is limited, but recent surveys indicate that, as in most other parts of Sub-Saharan Africa, *An. funestus* and *An. gambiae* are the principal LF vectors [37; ST Shawa personal communication]. These species are also the main malaria vectors in Sub-Saharan Africa. As Zambia is one of the countries in this region that has received relatively high bed net coverage and coverage of indoor residual spraying for malaria control in recent years [38], it cannot be excluded that these activities to some extent could have impacted the LF prevalences in some of the studied areas.

Identifying the ecological correlates of LF presence and exploring its environmental distribution in Zambia is an important step required to produce accurate and reliable maps for geographically targeted and cost-effective intervention. Here a machine learning approach, that allows flexible modeling and exploration of potential complex associations between infection presence and environmental predictor variables in geographical space, was applied. This approach allowed visualization of the ‘ecological space’ for occurrence of LF at different levels of infection prevalence, and provided new insights as to how environmental variables may functionally influence the LF parasite-vector-host bioescone in Zambia.

Of note it was found that the general distribution of LF (≥5%) in Zambia appeared to be associated with human modified land areas, as indicated by the strong association with croplands and the Human Influence Index. These areas may sustain habitat-types that are particularly suitable breeding areas for the main vector mosquito species in Zambia (*Anopheles gambiae* and *A. funestus*), and it is biological intuitive that the parasite is found in areas where the human host resides. It may, however, also partly be a reflection of a sampling bias towards (more densely) populated areas. Climatic factors on the other hand, were not important in model 1, suggesting that climate *per se* may play a smaller role in determining the general distribution of LF in Zambia.

The distribution of medium to high levels of LF (model 2) on the other hand, was less associated with human influenced predictors (only 1.9% HII) and seemed to be more related to climatic factors, with daytime temperature variables being equally important to land cover as measured by contribution to model training gain (Table 3) The functional relationship with day time temperature was positive (Figure 2b–c), reaching a plateaux (maximum) at around 31°C in the rainy season and with a lower limit at around 22°C (rainy and hot/dry season). This corresponds well with the findings from experimental studies showing that only few microfilariae will penetrate the gut of the mosquito at temperatures below 22°C and only little or no development occurs [39]–[42]. The rate of development then increases with rising temperatures, becoming optimal around 30°C [39]. Hereafter, the yield of infective larvae decreases due to increased filarial larval mortality [42], [43] and lower survival rate of infective mosquitoes [41]. It also corresponds well to the findings from continental scale studies of the distribution of LF in Africa. For example Lindsay and Thomas [9], who found that the temperatures at sites with presence of microfilaraemic individuals across Africa lie within the range between 22 to 30 degrees, and Slater and Michael [18] who found that the most suitable range for LF transmission across Africa lies between 25°C and 32.5°C (mean maximum temperature).

Besides suitable temperature ranges, water availability for mosquito breeding is a prerequisite for LF transmission. Rainfall however, did not contribute much to either models, and hence does not seem to be an important limiting factor for the distribution of LF in Zambia. However, distance from nearest permanent surface water body had the most information not present in the other variables in the models, and hence (together with land cover and temperature) appear to be an important determinant of LF distribution in Zambia.

Similar environmental information as applied in the current study was recently used to predict the distribution and risk of malaria across Zambia [44], although applying a different modeling approach (Bayesian geostatistical modeling). Given that LF in Zambia is transmitted by the same vector mosquito species with the same ecological requirements as malaria, a certain similarity between the distributions of the two infections is to be expected. A visual comparison of the two maps indicate areas of co-inciding high risk in the low-lying floodplains and valleys surrounding Luangwa River, on the border between Northern and Eastern Provinces and in eastern parts of Lusaka Province. An area of medium-high risk malaria is also predicted in the floodplain areas in Western Province (Zambezi River floodplains), although this is much more confined than that of the relatively large area predicted for LF in this part of the country. The biggest difference between the maps is the general high malaria risk predicted in large parts of Northern Province, where LF (at medium-high prevalence levels) is predicted to be less widespread. Similar patterns of contrasting spatial distributions of LF and malaria has also been observed in Uganda [45] and in some West African countries [10].

The present study has provided new and unexpected knowledge indicating widespread occurrence of LF in Zambia. It has moreover outlined its approximate geographical distribution, pointed to specific areas with high prevalence, and identified important environmental factors affecting its presence at various prevalence levels. This information will all be useful for planning and implementation of control of LF as a public health problem. In fact, the Ministry of Health in Zambia initiated mass drug administration in Kalabo District in late 2012, based on the findings from the field surveys reported in this paper, and it is planned to scale up this activity across the country in the next few years.

Although the applied modeling approach has proven useful to explore ecological correlates of LF and visualize environmentally suitable areas across unsurveyed areas in Zambia, it is important to stress that the resultant maps do not depict predicted prevalence: they show the relative probabilities of presence of the parasite-vector-host biocoenose. Given the relatively low correlation between these values and actual LF prevalence at sites, care should be taken not to interpret the maps as prevalence prediction maps. For this purpose, the full range of information in the survey data (i.e age and gender) also known to substantially influence LF prevalence/infection status, should be taken into consideration. Hence, a logical next step will be to build on the findings here and include individual level demographic data in a Bayesian geostatistical prediction model. Such an approach will allow an estimation of LF prevalence at unsurveyed locations, along with number of people at risk according to age and gender as done for instance for LF in Uganda [45], which would be particularly useful for further improved geographically targeted and cost-effective intervention.

## Supporting Information

Checklist S1 **STROBE checklist.**
(DOC)

Figure S1 **Spatial distribution of a selection of environmental predictors in Zambia.** The climatic factors were summarized over the survey period and according to climatic seasons in Zambia (NDVI; Normalized Difference Vegetation Index, LST; Land Surface Temperature. HII; Human Influence Index).
(TIF)

Methods S1 **MaxEnt modelling in brief.**
(DOCX)

Table S1 **Study location details.**
(DOCX)

## References

[1] Buckley JJC (1946) A helminthological survey in Northern Rhodesia. J Helminthol 21: 111–174.

[2] Barclay R (1971) Filariasis in Luangwa basin. Med J Zambia 5: 201–203.

[3] Hira PR (1975) Bancroftian filariasis in Zambia. Ann Trop Med Parasitol 69: 521–522.

[4] Hira PR (1976) Bancroftian filariasis. An autochthonous case in Zambia. Med J Zambia 10: 160–163. 1052106

[5] Hira PR (1977) *Wuchereria bancrofti*: The staining of the microfilarial sheath in giemsa and haematoxylin for diagnosis. Med J Zambia 11: 93–96. 919785

[6] Kaile T (1998) Filariasis presenting as pyrexia of unknown origin in a young Zambian adult: a case report. Zambian J Med Health Sci 2: 2–3.

[7] Matondo AS, Lungu AG (1998) Lymphatic filariasis in a Zambian woman: A case report. Zambian J Med Health Sci 2: 55–56.

[8] World Health Organization (2000) Operational guidelines for rapid mapping of bancroftian filariasis in Africa (WHO/CDS/CPE/CEE/2000.9). Geneva: WHO.

[9] Lindsay SW, Thomas CJ (2000) Mapping and estimating the population at risk from lymphatic filariasis in Africa. Trans R Soc Trop Med Hyg 94: 37–45. 10748895 10.1016/s0035-9203(00)90431-0

[10] Kelly-Hope L, Diggle PJ, Rowlingson BS, Gyapong JO, Keylem D, et al (2006) Short communication: Negative spatial association between lymphatic filariasis and malaria in West Africa. Trop Med Int Health 11: 129–135. 16451336 10.1111/j.1365-3156.2005.01558.x

[11] WHO (2011) Monitoring and epidemiological assessment of mass drug administration in the global programme to eliminate lymphatic filariasis: a manual for national elimination programmes (WHO/HTM/NTD/PCT/2011.4). Geneva: World Health Organization.

[12] Hijmans RJ, Cameron SE, Parra JL, Jones PG, Jarvis A (2005) Very high resolution interpolated climate surfaces for global land areas. Int J Climatol 25: 1965–1978.

[13] Sanderson EW, Jaiteh M, Levy MA, Redford KH, Wannebo AV, et al (2002) The human footprint and the last of the wild. Bioscience 52: 891–904.

[14] Elith J, Phillips SJ, Hastie T, Dudik M, Chee YE, et al (2011) A statistical explanation of MaxEnt for ecologists. Diversity Distrib 17: 43–57.

[15] Phillips SJ, Anderson RP, Schapire RE (2006) Maximum entropy modeling of species geographic distributions. Ecol Model 190: 231–259.

[16] Elith J, Graham CH, Anderson RP, Dudik M, Ferrier S, et al (2006) Novel methods improve prediction of species' distributions from occurrence data. Ecography 29: 129–151.

[17] Elith J, Graham CH (2009) Do they? How do they? WHY do they differ? On finding reasons for differing performances of species distribution models. Ecography 32: 66–77.

[18] Slater H, Michael E (2012) Predicting the current and future potential distributions of lymphatic filariasis in Africa using maximum entropy ecological niche modelling. PloS One 7: e32202. 22359670 10.1371/journal.pone.0032202 PMC3281123

[19] Stensgaard AS, Utzinger J, Vounatsou P, Hürlimann E, Schur N, et al (2013) Large-scale determinants of intestinal schistosomiasis and intermediate host snail distribution across Africa: does climate matter? Acta Trop 128: 378–390. 22142789 10.1016/j.actatropica.2011.11.010

[20] Guisan A, Thuiller W (2005) Predicting species distribution: offering more than simple habitat models. Ecol Lett 8: 9931009. 10.1111/j.1461-0248.2005.00792.x 34517687

[21] Pearce JL, Boyce MS (2006) Modelling distribution and abundance with presence-only data. J Appl Ecol 43: 405–412.

[22] Elith J, Leathwick JR (2009) Species distribution models: ecological explanation and prediction across space and time. Annu Rev Ecol Evol Syst 40: 677–697.

[23] Michael E, Malecela Mn, Zervos M, Kazura JW (2008) Global eradication of lymphatic filariasis: the value of chronic disease control in parasite elimination programmes. PLoS One 3: e2936. 18698350 10.1371/journal.pone.0002936 PMC2490717

[24] Hastie T, Tibshirani R, Friedman JH (2009) The elements of statistical learning: data mining, interference, and prediction, 2^nd^ edn. Springer-Verlag, New York.

[25] Wisz MS, Hijmans RJ, Li J, Peterson AT, Graham CH, Guisan A (2008) Effects of sample size on the performance of species distribution models. Divers Distrib 14: 763–773.

[26] Phillips SJ, Dudik M (2008) Modeling of species distributions with Maxent: new extensions and a comprehensive evaluation. Ecography 31: 161–175.

[27] Liu C, Berry PM, Dawson T, Pearson RG (2005) Selecting thresholds of occurrence in the prediction of species distributions. Ecography 28: 385–393.

[28] Brabin L (1990) Sex differentials in susceptibility to lymphatic filariasis and implications for maternal child immunity. Epidemiol Infec 105: 335–353. 2209738 10.1017/s0950268800047932 PMC2271898

[29] Onapa AW, Simonsen PE, Pedersen EM, Okello DO (2001) Lymphatic filariasis in Uganda: baseline investigations in Lira, Soroti and Katakwi districts. Trans R Soc Trop Med Hyg 95: 161–167. 11355548 10.1016/s0035-9203(01)90145-2

[30] Simonsen PE, Meyrowitsch DW, Jaoko WG, Malecela-Lazaro MN, Mukoko D, et al (2002) Bancroftian filariasis infection, disease and specific antibody response patterns in a high and a low endemicity community in East Africa. Am J Trop Med Hyg 66: 550–559. 12201589 10.4269/ajtmh.2002.66.550

[31] Roberts CJ, McKeag A, Gelfand M (1971) *A. perstans* and *W. bancrofti*. A filarial survey in a game reserve in the Zambezi Valley. Central African J Med 17: 101–102. 5112594

[32] Roberts CJ, Whitehall J, Gelfand M (1973) *W. bancrofti* in Kenyemba area. Central African J Med 19: 13–14. 4712840

[33] Azevedo JF, Pinhão R, Meira M, Gardette M (1969) Bancroftian and malayan filariasis in overseas Portuguese territories. An Esc Nac Saude Publica Med Trop (Lisb) 3: 3–9. 5404999

[34] Ngwira BMM, Tambala P, Perez AM, Bowie C, Molyneux DH (2007) The geographical distribution of lymphatic filariasis in Malawi. Filaria J 6: 12. 18047646 10.1186/1475-2883-6-12 PMC2233609

[35] Kelly-Hope LA, Thomas BC, Bockarie MJ, Molyneux DH (2011) Lymphatic filariasis in the Democratic Republic of Congo; micro-stratification overlap mapping (MOM) as a prerequisite for control and surveillance. Parasit Vectors 4: 178. 21923949 10.1186/1756-3305-4-178 PMC3183006

[36] Simonsen PE, Onapa AW, Asio AM (2011) *Mansonella perstans* filariasis in Africa. Acta Trop 120S: 109–120. 10.1016/j.actatropica.2010.01.014 20152790

[37] Shawa ST, Mwase ET, Pedersen EM, Simonsen PE (2013) Lymphatic filariasis in Luangwa District, South-East Zambia. Parasit Vectors 6: 299. 24499525 10.1186/1756-3305-6-299 PMC3853755

[38] Kelly-Hope LA, Molyneux DH, Bockarie MJ (2013) Can malaria vector control accelerate the interruption of lymphatic filariasis transmission in Africa; Capturing a window of opportunity? Parasit Vectors 6: 39. 23433078 10.1186/1756-3305-6-39 PMC3599698

[39] Rao SS, Iyengar MOT (1930) Studies on the influence of season on the development of *Filaria bancrofti* in *Culex fatigans* Indian. J Med Res 17: 759–781.

[40] Omori N (1958) Experimental studies on the role of the house mosquito, *Culex pipiens pallens*, in the transmission of bancroftian filariasis 5. On the distribution of infective larvae in the mosquito and the effects of parasitism of filariae upon the host insect. Nagasaki Med J 33: 143–155.

[41] Nakamura Y (1965) Experimental studies on the role of *Aedes togoi* in the transmission of bancroftian filariasis III. Longevity of adult mosquitoes fed on sugar solutions at various constant temperatures. Endemic Dis Bull (Nagasaki) 6: 113–124.

[42] Lardeux F, Cheffort J (1997) Temperature threshold and statistical modelling of larval *Wuchereria bancrofti* (Filariidea: Onchocercidae) developmental rates. Parasitol 114: 123–124. 10.1017/s0031182096008359 9051921

[43] Brunhes J (1969) Nouvelles donnees sur les vecteurs de *Wuchereria bancrofti* a Madagascar. Influence de la temperature sur la vitesse de development du parasite et le taux d′ infection du vecteur. Bull World Health Organ 40: 763–9. 5307236 PMC2554500

[44] Riedel N, Vounatsou P, Miller JM, Gosoniu L, Chizema-Kawesha E, et al (2010) Geographical patterns and predictors of malaria risk in Zambia: Bayesian geostatistical modelling of the 2006 Zambia national malaria indicator survey (ZMIS). Malaria J 9: 37. 10.1186/1475-2875-9-37 PMC2845589 20122148

[45] Stensgaard AS, Vounatsou P, Onapa AW, Simonsen PE, Pedersen EM, et al (2011) Bayesian geostatistical modeling of malaria and lymphatic filariasis infections in Uganda: predictors of risk and geographical patterns of co-endemicity. Malaria J 10: 298. 10.1186/1475-2875-10-298 PMC3216645 21989409

