# Supplementary figures and images for: Mapping the Geographical Distribution of Lymphatic Filariasis in Zambia
Source: PLoS Negl Trop Dis. 2014 Feb 20;8(2):e2714. doi: 10.1371/journal.pntd.0002714 (PMC3930513; doi:10.1371/journal.pntd.0002714)

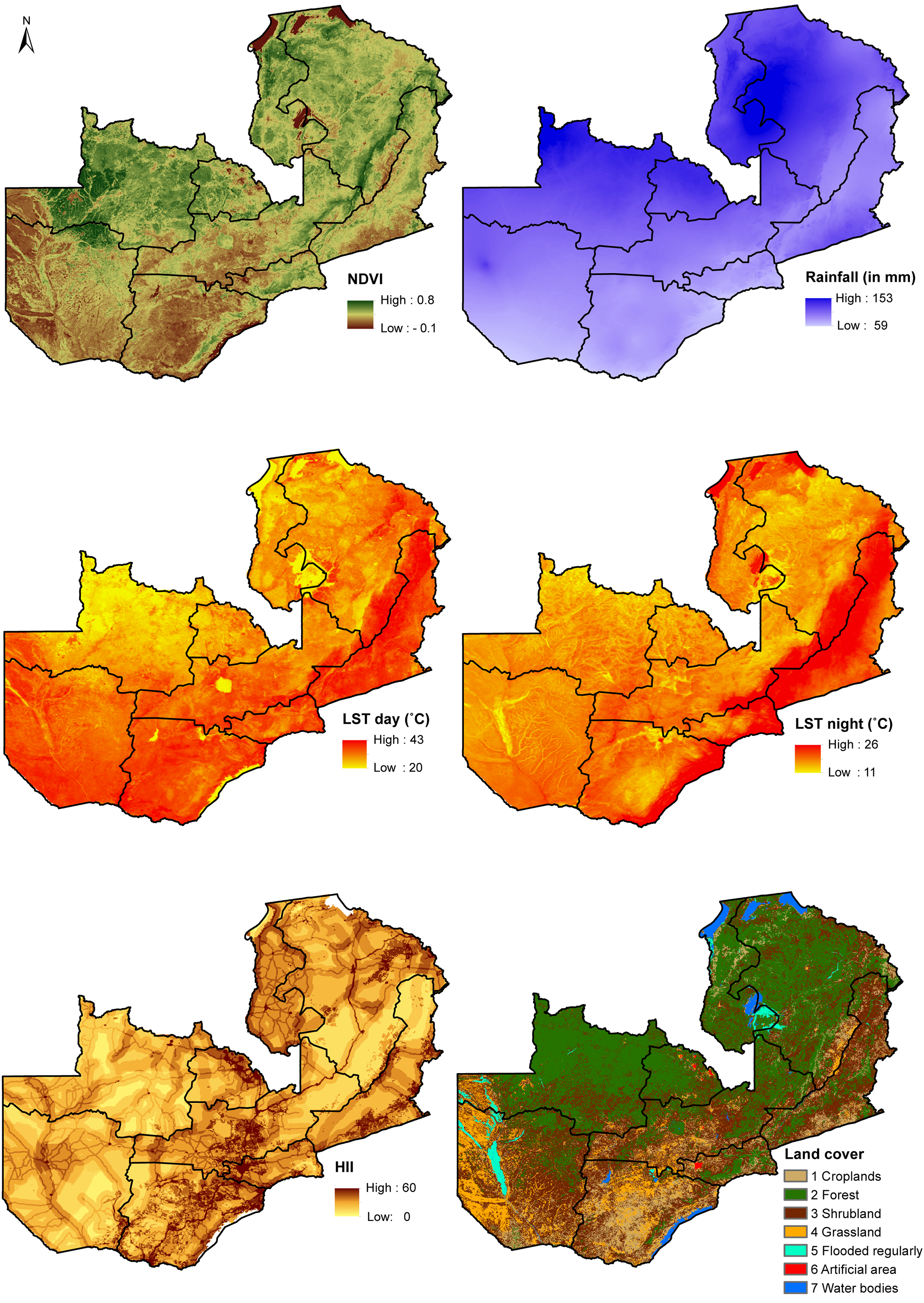

Supplement: Figure S1 — Spatial distribution of a selection of environmental predictors in Zambia. The climatic factors were summarized over the survey period and according to climatic seasons in Zambia (NDVI; Normalized Difference Vegetation Index, LST; Land Surface Temperature. HII; Human Influence Index). (TIF) [file pntd.0002714.s002.tif]
