# Supplementary material for: Mapping the Geographical Distribution of Lymphatic Filariasis in Zambia
Source: PLoS Negl Trop Dis. 2014 Feb 20;8(2):e2714. doi: 10.1371/journal.pntd.0002714 (PMC3930513; doi:10.1371/journal.pntd.0002714)
Supplement: Methods S1 — MaxEnt modelling in brief. (DOCX) [file pntd.0002714.s003.docx]

**Methods S1: MaxEnt modeling in brief**

**How it works:**

MaxEnt is a general-purpose machine learning method for characterizing probability distributions from incomplete information (Philips et al., 2006). To estimate the probability distribution that define a species’ distribution across a given study area, MaxEnt starts by assuming the probability is perfectly uniform in geographic space. A constraint component then ensures that the estimated distribution must ‘agree with everything that is known’ (Harte et al., 2010): i.e that which can be inferred from the environmental conditions at the observed presence locations. The constraints are expressed in terms of simple functions of the environmental variables, called features (Philips and Dudik, 2008). As many distributions potentially fulfill these constraints, the principle of maximum entropy is used to choose among the many potential probability distributions that fit the data.

MaxEnt does not require absence data for the species being modeled; instead it uses background environmental data for the entire study area (in our case, the whole of Zambia). This background sample is taken from a landscape of locations that are considered to be a priori equally likely to contain the species (default is a random sample of 10,000 background points). The presence/absence is treated as unknown at these locations, and MaxEnt contrasts the features at the presence locations to those in the background sample (Merow et al., 2013).

During a model run, MaxEnt conducts an optimization routine that iteratively improves model fit, in machine learning referred to as the gain (Philips et al., 2006). The gain is a penalized likelihood function, closely related to a likelihood (deviance) statistic, that MaxEnt maximizes. Exponentiating the gain gives the likelihood ratio of an average presence to an average background point (Elith et al., 2011).

**Evaluating environmental predictor importance:**

While the MaxEnt model is being trained, track is kept of which environmental predictors make the greatest contribution to the overall model gain. Through iterations MaxEnt increases the gain of the model by modifying the coefficient for a single feature based on input environmental data (Philips et al., 2006). At the end of the run, the program assigns contributions in percentages of each environmental variable to the overall model gain. These are the values represented in Table 3 in the current study.

The importance of the environmental predictors can also be evaluated using a jackknife procedure, where by each variable is excluded in turn, creating a model with the remaining variables, and furthermore a model using each variable in isolation. A variable that used in isolation achieves almost no gain is not (in itself) useful for estimating the distribution of the species, while variables exhibiting high gains allow a reasonably good fit to the training data. Conversely, a variable that shows considerably large decreases in model gain when omitted from the full model contains a substantial amount of useful information that is not contained in the other variables. In this study such a variable was the ‘distance to surface water bodies’, although this variable did not contribute a high proportion of total model training gain as expressed in percentage contribution.

**References**

Elith J, Phillips SJ, Hastie T, Dudik M, Chee YE, et al (2011) A statistical explanation of MaxEnt for ecologists. Diversity Distrib 17: 43-57.

Harte J (2011). Maximum entropy and ecology: theory of abundance, distributions, and energetics. Oxford: Oxford University Press.

Merow C, Smith M J, Silander Jr JA (2013). A practical guide to MaxEnt for modeling species’ distributions: what it does, and why inputs and settings matter. – Ecography 36: 1-12.

Phillips SJ, Anderson RP, Schapire RE (2006) Maximum entropy modeling of species geographic distributions. Ecol Model 190: 231-259.

Phillips SJ, Dudik M (2008) Modeling of species distributions with Maxent: new extensions and a comprehensive evaluation. Ecography 31: 161-175.
