# Supplementary material for: Mapping the Geographical Distribution of Lymphatic Filariasis in Zambia
Source: PLoS Negl Trop Dis. 2014 Feb 20;8(2):e2714. doi: 10.1371/journal.pntd.0002714 (PMC3930513; doi:10.1371/journal.pntd.0002714)
Supplement: Table S1 — Study location details. (DOCX) [file pntd.0002714.s004.docx]

**Table S1: Study location details.** Year of survey, longitude and latitude for the 108 study sites.

| **Site no.** | **Province** | **District** | **Village/ Chiefdom/Site** | **Year of survey** | **Longitude** | **Latitude** |
| --- | --- | --- | --- | --- | --- | --- |
|  |  |  |  |  |  |  |
| 1 | Central | Mkushi | Masansa | 2009 | -13.94523 | 29.33724 |
| 2 |  | Kapiri Mposhi | Tazara | 2009 | -13.95684 | 28.68075 |
| 3 |  | Chibombo | Chibombo | 2009 | -14.66336 | 28.07662 |
| 4 |  | Kabwe | Kasanda | 2009 | -14.45546 | 28.42628 |
| 5 |  | Mumbwa | Keezwa | 2009 | -15.39011 | 27.45323 |
| 6 |  | Serenje | Mulilima | 2005 | -13.21955 | 29.54961 |
| 7 |  | Serenje | Muchinka | 2005 | -12.56241 | 30.26406 |
| 8 |  | Serenje | Mapepala | 2005 | -12.41935 | 30.08226 |
| 9 | Copperbelt | Mpongwe | Mwanankonesha/Lesa | 2003 | -13.59260 | 28.40910 |
| 10 |  | Mpongwe | Machiya | 2003 | -13.64184 | 27.60477 |
| 11 |  | Mpongwe | Mwinuna | 2003 | -13.83561 | 27.83713 |
| 12 |  | Masaiti | Fiwale Mission | 2010 | -13.20905 | 28.71721 |
| 13 |  | Ndola | Chipulukusu | 2010 | -12.94434 | 28.65772 |
| 14 |  | Luanshya | Mpatamatwe | 2010 | -13.09887 | 28.31039 |
| 15 |  | Kitwe | Buchi | 2010 | -12.79509 | 28.20923 |
| 16 |  | Chililabombwe | Kawama | 2010 | -12.32960 | 27.85828 |
| 17 |  | Lufwanyama | St. Joseph Mission | 2010 | -12.89162 | 28.01319 |
| 18 |  | Kalulushi | Chibuluma | 2010 | -12.85760 | 28.13719 |
| 19 |  | Mufulira | Lwansobe | 2010 | -12.51235 | 28.18865 |
| 20 |  | Chingoloa | Chawama | 2010 | -12.55812 | 27.84022 |
| 21 | Eastern | Chadiza | Nsadzu | 2010 | -14.04126 | 32.20207 |
| 22 |  | Chipata | Madzimoyo | 2010 | -13.69948 | 32.50237 |
| 23 |  | Mambwe | Masumba | 2010 | -13.20920 | 31.94050 |
| 24 |  | Katete | Katete Urban | 2010 | -14.09109 | 32.06440 |
| 25 |  | Nyimba | Chipembe | 2010 | -14.53558 | 31.01949 |
| 26 |  | Petauke | Mumba | 2010 | -14.47908 | 31.31067 |
| 27 |  | Lundazi | Zumwanda | 2005 | -12.25324 | 33.03032 |
| 28 |  | Lundazi | Nkhanga | 2005 | -12.09161 | 33.02249 |
| 29 |  | Lundazi | Mwase-Lundazi | 2005 | -12.24413 | 33.20236 |
| 30 |  | Chama | Chipundu-Kambombo | 2003 | -11.08391 | 33.09528 |
| 31 |  | Chama | Mbubeni-Tembwe | 2003 | -11.36027 | 32.88935 |
| 32 |  | Chama | Chitunda-Chikwa | 2003 | -11.68203 | 32.77480 |
| 33 | Luapula | Chiengi | Puta | 2009 | -8.68780 | 29.14726 |
| 34 |  | Nchelenge | Nchelenge | 2009 | -9.34539 | 28.73591 |
| 35 |  | Kawambwa | Mukamba | 2009 | -11.26611 | 29.05390 |
| 36 |  | Mwense | Lubunda | 2009 | -10.31213 | 28.67342 |
| 37 |  | Mwense | Musangu | 2009 | -10.24702 | 28.64948 |
| 38 |  | Mwense | Lukwesa | 2009 | -10.16891 | 28.63603 |
| 39 |  | Mansa | Mabumba | 2009 | -11.15587 | 29.03134 |
| 40 |  | Samfya | Mandubi | 2009 | -11.17489 | 29.57393 |
| 41 |  | Milenge | Milenge East 7* | 2009 | -12.41640 | 29.48487 |
| 42 | Lusaka | Lusaka | Chipata | 2009 | -13.62157 | 28.77019 |
| 43 |  | Chongwe | Rufunsa | 2009 | -13.71706 | 29.55876 |
| 44 |  | Kafue | Chanyanya Harbour | 2005 | -15.41178 | 28.00257 |
| 45 |  | Kafue | Kanjawa | 2005 | -15.40393 | 28.06422 |
| 46 |  | Kafue | Tukunta | 2005 | -15.40096 | 28.01058 |
| 47 |  | Luangwa | Kavalamanja-Mphuka | 2003 | -15.61524 | 30.26296 |
| 48 |  | Luangwa | Janeiro-Mphuka | 2003 | -15.42445 | 30.30612 |
| 49 |  | Luangwa | Chitope-Mburuma | 2003 | -15.18772 | 30.21813 |
| 50 | Northern | Luwingu | Nsombo | 2009 | -10.81509 | 29.93965 |
| 51 |  | Chilubi | Chaba | 2009 | -10.97870 | 30.08575 |
| 52 |  | Kaputa | Kalaba | 2009 | -8.41696 | 29.87507 |
| 53 |  | Mporokoso | Chishamwanba | 2009 | -9.34672 | 30.09380 |
| 54 |  | Mpulungu | Mpulungu | 2009 | -8.76310 | 31.11469 |
| 55 |  | Isoka | Kampumbu | 2009 | -10.25592 | 30.00820 |
| 56 |  | Nakonde | Shemu | 2009 | -9.52742 | 32.90198 |
| 57 |  | Mungwi | Mumba | 2009 | -10.38782 | 31.83226 |
| 58 |  | Kasama | Munkonge | 2009 | -10.45463 | 30.67731 |
| 59 |  | Mpika | Nabwalya | 2009 | -12.41859 | 31.97807 |
| 60 |  | Mpika | Mpepo | 2009 | -11.08051 | 31.11665 |
| 61 |  | Mbala | Chilundumusi | 2003 | -9.16931 | 32.04720 |
| 62 |  | Mbala | Mwamba | 2003 | -8.91812 | 31.62919 |
| 63 |  | Mbala | Chiungu-Zombe | 2003 | -8.62399 | 31.28272 |
| 64 |  | Chinsali | Ilondola-Nkula | 2003 | -10.42810 | 31.48184 |
| 65 |  | Chinsali | Nkweto | 2003 | -10.15411 | 32.17928 |
| 66 |  | Chinsali | Mulanga** | 2003 | -10.59487 | 32.09027 |
| 67 | North-Western | Mwinilunga | Kalene Mission | 2009 | -11.17560 | 24.18807 |
| 68 |  | Solwezi | Solwezi Urban | 2009 | -12.18743 | 26.39735 |
| 69 |  | Solwezi | Lumwana East | 2009 | -12.27158 | 25.65627 |
| 70 |  | Kasempa | Kasempa Urban | 2009 | -13.45834 | 25.83262 |
| 71 |  | Mufumbwe | Boma | 2009 | -13.14079 | 25.00708 |
| 72 |  | Kabompo | Kapompo | 2009 | -13.59622 | 24.20558 |
| 73 |  | Chavuma | Chiyeke | 2009 | -13.07354 | 22.73300 |
| 74 |  | Zambezi | Kucheka | 2005 | -13.46644 | 22.54022 |
| 75 |  | Zambezi | Mukandankunda*** | 2005 | -13.23639 | 23.03836 |
| 76 |  | Zambezi | Chinyingi-Ndungu | 2005 | -13.21526 | 23.00685 |
| 77 | Southern | Livingstone | Lubuyu | 2010 | -17.85350 | 25.88339 |
| 78 |  | Kazungula | Makunka | 2010 | -17.53705 | 25.64226 |
| 79 |  | Kalomo | Namiyanga | 2010 | -17.06452 | 26.48676 |
| 80 |  | Monze | Njola Mwanza | 2010 | -16.21828 | 27.71350 |
| 81 |  | Itezhitezhi | Itezhitezhi Urban | 2010 | -15.73970 | 26.03265 |
| 82 |  | Gweembe | Munyumbwe | 2010 | -16.64642 | 27.77721 |
| 83 |  | Siavonga | Siavonga District | 2010 | -16.53838 | 28.70300 |
| 84 |  | Namwala | Muchila | 2010 | -16.23548 | 26.59770 |
| 85 |  | Namwala | Chitongo | 2010 | -16.03740 | 26.93000 |
| 86 |  | Mazabuka | Cheeba | 2011 | -15.47190 | 28.20432 |
| 87 |  | Choma | Simachenga-Singani | 2003 | -16.46147 | 27.15029 |
| 88 |  | Choma | Macha | 2003 | -16.41929 | 26.78837 |
| 89 |  | Choma | Moyo | 2003 | -16.98874 | 27.31673 |
| 90 |  | Sinazongwe | Sinazeze | 2003 | -17.08706 | 27.24285 |
| 91 |  | Sinazongwe | Sinazongwe | 2003 | -17.14890 | 27.27024 |
| 92 |  | Sinazongwe | Mwemba | 2003 | -17.27533 | 27.17437 |
| 93 | Western | Kaoma | Mangango Mission | 2009 | -14.65716 | 24.51148 |
| 94 |  | Kaoma | Mayukwayukwa 1 | 2009 | -14.53842 | 24.20901 |
| 95 |  | Lukulu | Silembe**** | 2009 | -14.26675 | 23.25560 |
| 96 |  | Mongu | Nalikwanda***** | 2009 | -15.48305 | 23.65678 |
| 97 |  | Shangombo | Nangweshi | 2009 | -16.39591 | 23.32716 |
| 98 |  | Mongu | Sefula–Namutwe | 2009 | -15.38395 | 23.18617 |
| 99 |  | Kalabo | Maunyambo | 2009 | -14.99279 | 22.66542 |
| 100 |  | Sesheke | Mulundamo | 2005 | -16.51667 | 24.70000 |
| 101 |  | Sesheke | Malabwe | 2005 | -16.86667 | 25.11667 |
| 102 |  | Sesheke | Sazibilo | 2005 | -16.66667 | 24.95000 |
| 103 |  | Senanga | Itufa-Lityamba | 2005 | -15.51302 | 23.18444 |
| 104 |  | Senanga/Shangombo | Kanja/Nangweshi | 2005 | -16.23436 | 23.19365 |
| 105 |  | Senanga | Kaunga Lueti | 2005 | -16.04120 | 23.01518 |
| 106 |  | Kalabo | Nalubutu Sishekanu | 2003 | -14.54937 | 22.46612 |
| 107 |  | Kalabo | Kaonga Sikongo | 2003 | -15.02092 | 22.03246 |
| 108 |  | Kalabo | Lwandamo Lutwi | 2003 | -15.16852 | 22.34050 |

* Milenge East 7 & Changwe Lungo

** Mulanga-Chibesakunda

*** Mukandankunda-Ishindi

**** Silembe Kalambwe-Imenda

***** Nalikwanda–Singonda
